# Mixed Effects of Soil Compaction on the Nitrogen Cycle Under Pea and Wheat

**DOI:** 10.3389/fmicb.2021.822487

**Published:** 2022-03-07

**Authors:** Manon Longepierre, Rafaela Feola Conz, Matti Barthel, David Bru, Laurent Philippot, Johan Six, Martin Hartmann

**Affiliations:** ^1^Sustainable Agroecosystems, Department of Environmental Systems Science, ETH Zürich, Zurich, Switzerland; ^2^Department of Agroecology, University of Bourgogne Franche-Comté, INRAE, AgroSup Dijon, Dijon, France

**Keywords:** soil compaction, nitrogen cycling, soil microbiome, *amoA* genes, *nirS* and *nirK* genes, *nosZ* clade I and II genes, pea (*Pisum sativum*), wheat (*Triticum dicoccum*)

## Abstract

Soil compaction caused by highly mechanized agriculture can constrain soil microbial diversity and functioning. Physical pressure on the soil decreases macropores and thereby limits oxygen diffusion. The associated shift from aerobic to anaerobic conditions can reduce nitrification and promote denitrification processes, leading to nitrogen (N) losses and N depletion that affect plant productivity. High soil moisture content during trafficking can exacerbate the negative effects of soil compaction. However, the extent to which soil moisture amplifies the effects of compaction on the soil microbiome and its control over N cycling is not well understood. Using a controlled greenhouse experiment with two different crops (pea and wheat), we compared the effects of compaction at three different soil moisture levels on soil physicochemical properties, microbial diversity, and the abundance of specific N species and quantification of associated microbial functional groups in the N cycle. Soil compaction increased bulk density from 15% (light compaction) to 25% (severe compaction). Compaction delayed germination in both crops and reduced yield by up to 60% for pea and 40% for wheat. Compaction further induced crop-specific shifts in microbial community structures. After compaction, the relative abundance of denitrifiers increased along with increased nitrate (NO_3_^–^) consumption and elevated nitrous oxide (N_2_O) concentrations in the soil pores. Conversely, the relative abundance of nitrifiers remained stable under compaction, but potentially decelerated nitrification rates, resulting in ammonium (NH_4_^+^) accumulation in the soil. This study showed that soil compaction effects are proportional to the initial soil moisture content, which could serve as a good indicator of compaction severity on agricultural fields. However, the impact of soil compaction on crop performance and on microbial communities and functions associated with the N cycle were not necessarily aligned. These findings demonstrate that not only the soil physical properties but also various biological indicators need to be considered in order to provide more precise recommendations for developing sustainable farming systems.

## Introduction

Soil compaction is a major problem in modern agriculture (Hamza and Anderson, 2005; Nawaz et al., 2013) and has been recognized by the Food and Agriculture Organization (FAO) as one of the main threats to soil functionality (FAO, 2015). Heavy machineries, used during tillage, sowing, fertilizer application, and harvesting, are responsible for soil compaction (Hamza and Anderson, 2005). However, the degree of compaction is not only influenced by machine weight, tire pressure, and the number of passes but also by the initial intrinsic characteristics of the soil such as its texture and moisture level (Défossez et al., 2003; Shah et al., 2017).

Compaction leads to a decrease in soil porosity and aggregation as well as an increase in soil bulk density and penetration resistance (Pagliai et al., 2003; Schäffer et al., 2008). These changes in soil structure can reduce water infiltration and lead to increased water run-off (Shah et al., 2017). Simultaneously, oxygen infiltration and diffusion into the soil can decrease and thus foster anoxic conditions (Nawaz et al., 2013). As a result, soil compaction can decrease microbial biomass (Pupin et al., 2009; Weisskopf et al., 2010) and alter microbial community structures (Hartmann et al., 2014; Longepierre et al., 2021). For example, bacteria capable of anaerobic respiration, such as methanogens and denitrifiers, are promoted (Li et al., 2004; Hartmann et al., 2014; Longepierre et al., 2021), resulting in reduced carbon dioxide production (Novara et al., 2012) and increased methane and nitrous oxide emissions (Hartmann et al., 2014).

The shift from more aerobic toward more anaerobic conditions with soil compaction can affect the nitrogen (N) cycle, which consists of a combination of aerobic and anaerobic pathways mediated by different microbial groups, including archaea, bacteria, and fungi (Hayatsu et al., 2008). Nitrification is the aerobic oxidation of ammonium (NH_4_^+^) into nitrite (NO_2_^–^) and nitrate (NO_3_^–^). The first nitrification step is performed by ammonia-oxidizing bacteria (AOB) and ammonia-oxidizing archaea (AOA) (Hayatsu et al., 2008). Conversely, denitrification is an anaerobic reduction of nitrate (NO_3_^–^) into nitrite (NO_2_^–^), nitric oxide (NO), nitrous oxide (N_2_O), and molecular nitrogen (N_2_) (Hayatsu et al., 2008). In compacted soils, limited oxygen availability can reduce nitrification (Li et al., 2004; Tan and Chang, 2007; Siczek and Lipiec, 2011) and increase denitrification activities (Li et al., 2004; Weisskopf et al., 2010), ultimately leading to increased N_2_O emissions (Nawaz et al., 2013) and fertilizer N losses (Skiba and Smith, 2000; Oertel et al., 2016).

Since soil moisture content is one of the main drivers of compaction severity, all compaction effects previously listed can be augmented under wet soil (Hartmann et al., 2014). However, even though there is a good understanding of the soil compaction effects on soil physical properties (Gysi et al., 1999), there is still a lack of comparable knowledge on soil microbial communities and functions associated with the N cycle.

The aim of this study was to investigate the impact of soil compaction at different soil moisture levels on soil physicochemical properties, soil microbial diversity, the abundance of specific functional groups within the N cycle, and its associated products, and ultimately its impact on plant performance. Soil microcosms with three different soil moisture conditions were established to evaluate the impact of compaction on the concentration of different N compounds, including NH_4_^+^, NO_3_^–^, and N_2_O in the soil. At the same time, the abundance of the microbial functional groups involved in N cycling, such as nitrifying and denitrifying bacteria, were evaluated using quantitative PCR (qPCR) assays, combined with an overall assessment of the shifts in the bacterial community structure using a metabarcoding approach of the 16S ribosomal RNA (rRNA) gene. We expected that soil compaction levels and effects on the plant–soil system should be proportional to the initial soil moisture content during the compaction event. We hypothesized that soil compaction would be associated with a reduction in plant biomass, an accumulation in NH_4_^+^ due to reduced nitrification (approximated by measuring nitrifier abundance), and a depletion in soil NO_3_^–^ due to increased denitrification (approximated by measuring denitrifier abundance).

## Materials and Methods

### Experimental Design

The soil used for the experiment was composed of 4.4% clay, 46.8% silt, and 48.8% sand and can be classified as a “Sandy Loam” based on the USDA–NRCS classification system. Sandy loams are among the most susceptible to compaction because of their high silt content and unstable microporous structure (Richard et al., 2001; Shah et al., 2017). The soil was sieved at 8 mm and filled in 200 pots with a dimension of 16 cm diameter × 14 cm height. The pots, with the same treatment, were randomized in the greenhouse, so that spatial effects had limited influence on the analysis and were left undisturbed for 3 weeks to reach equilibrium. The soils in the pots were split into four conditions. The control pots were left uncompacted with an initial moisture content of 8.3% (Table 1). The other pots were compacted at three different levels of soil moisture (e.g., 8.2, 41.5, and 55.7%) but with the same pressure (200 kPa m^–2^) by using a hydraulic press (ENERPAC, Menomonee Falls, WI, United States) and a compression load cell (Brütsch/Rüegger Tools, Urdorf, Switzerland) in between the press and the soil (Table 1). The adjustment of the soil to different moisture levels prior to applying the compressive load was used to induce different severities of compaction as described previously (Hartmann et al., 2014). Under dry conditions, the compaction severity by a given pressure load is expected to be lower than by the same pressure load under more wet conditions. Hence, this setup simulates the more severe compaction events when driven on wet versus dry fields. The most severe compaction was obtained by watering the pots with 600 ml of water, and moderate compaction was obtained with 300 ml prior to applying the pressure load.

**TABLE 1:** Soil moisture levels at the time of compaction and respective treatment nomenclature.

Soil moisture (%)	Compaction pressure (kPa m^–2^)	Treatment
8.3 ± 0.4	0	Control
8.2 ± 0.3	200	Light compaction
41.5 ± 0.8	200	Moderate compaction
55.7 ± 0.8	200	Severe compaction

After compaction, the pots were split into two groups and sown with either pea (*Pisum sativum* cultivar ‘Blauwschokker’ from ProSpeciesRara, Switzerland) or wheat (*Triticum dicoccum* cultivar ‘Emmer’ from ProSpeciesRara, Switzerland). After sowing, pots were fertilized with liquid fertilizer (WUXAL, 8% N, 8% P_2_O_5_, 6% K, and a total of 9% of B, Cu, Fe, Mn, Mo, and Zn), adding the equivalent of 40 kg N ha^–1^. The experiment lasted for 2 months for pea and for 4 months for wheat. Plant growth conditions in the greenhouse were maintained at 22.5 ± 2.5°C and additional light from 8 a.m. to 8 p.m. Plants were irrigated on a regular basis. The destructive sampling of 40 pots (5 replicates × 4 compaction treatments × 2 crops) was performed at the same time interval when the pots were harvested for measuring plant shoot biomass, soil NH_4_^+^ and NO_3_^–^ concentrations, bulk density, soil moisture, the abundance of key microbial functional groups involved in the N cycle, and overall bacterial community structure.

To determine soil bulk density, a sample of the uppermost soil layer (0–5 cm depth) in each pot was collected periodically using a 100 cm^3^ cylinder and dried in an oven at 105°C overnight, followed by weighing the next day. Gravimetric soil moisture content was measured by collecting 10 g of the soil, drying it in an oven at 105°C overnight, and weighing it after 24 h.

### Plant Biomass

In all pots, four seeds were planted at the beginning of the experiment to increase the chance of having surviving seedlings of pea or wheat growing in the pots. After 1 week, only one random “healthy” plant was kept in all pots for each treatment condition. To measure plant biomass, the above-ground part of the plant was cut off and weighed immediately at the destructive sampling time points. Pea seeds were harvested, counted, and weighted after 2 months, whereas wheat ears were harvested, counted, and weighted after 4 months in order to estimate the productivity of the plant.

### Soil Pore CO_2_ and N_2_O Concentrations

During initial potting, a 10 cm hydrophobic capillary membrane segment (ø = 5.5 mm, ACCUREL^©^ PP; Membrana GmbH, Wuppertal, Germany) was placed in the soil at intermediate depth with a connection to an outlet port in order to sample for soil pore gas (Verhoeven et al., 2018). Soil pore gas sampling was done at 2 days before seeding (representing the initial compaction impact), as well as 1 week, 2 weeks, 1 month, 2 months, and 4 months after seeding and fertilizing. Samples were taken from each sampling port using a 20 ml disposable plastic syringe and transferred to a pre-evacuated 12 ml Exetainer (Labco Ltd., Lampeter, United Kingdom). Gas samples were subsequently analyzed for N_2_O and CO_2_ concentrations using gas chromatography (SCION 456-GC; Bruker, Billerica, MA, United States) calibrated with a suite of standards with the following concentrations: (1) 3,030 ppm CO_2_, 3.17 ppm N_2_O; (2) 249 ppm CO_2_, 1.2 ppm N_2_O; (3) 497.9 ppm CO_2_, 0.393 ppm N_2_O; (4) 0 ppm CO_2_, and 18.17 ppm N_2_O.

### Soil NH_4_^+^ and NO_3_^–^ Concentrations

NH_4_^+^ and NO_3_^–^ from each soil sample were extracted from 10 g wet soil with 2M KCl solution in a total volume of 50 ml and agitated at 250 rpm for 1 h. The soil–KCl solution was filtered with a 150 nm filter paper, and the clear solution was stored at −20°C until analysis. NH_4_^+^ and NO_3_^–^ were analyzed colorimetrically with a spectrophotometer v-1200 (VWR, Radnor, PA, United States) using the method outlined by Forster (1995) for NH_4_^+^ and the method outlined by Doane and Horwáth (2003) for NO_3_^–^. Briefly, the soil NH_4_^+^ concentration was measured on 800 μl of each sample with 200 μl of reagent A (0.05 g sodium nitroprusside, 13 g sodium salicylate, 10 g sodium citrate, and 10 g sodium tartrate dissolved in 100 ml Milli-Q-water) and 200 μl of reagent B (6 g sodium hydroxide dissolved in 100 ml water and 2 ml sodium hypochlorite). After 1 h of incubation at room temperature, the absorbance was read at 650 nm with a spectrophotometer. The standard curve was obtained by measuring the NH_4_^+^ concentration from standard solutions at concentrations of 0, 0.1, 0.2, 0.3, 0.4, 0.5, 1, 5, and 10 ppm NH_4_^+^. The soil NO_3_^–^ concentration was measured on 30 μl of each sample with 3,500 μl of 1:1 diluted reagent [200 ml of 0.5 M HCl, 0.5 g vanadium (III) chloride, 0.2 g sulphanilamide, and 0.01 g N (1-naphthyl) ethylenediamine dihydrochloride]. After 8 h, the absorbance was read at 540 nm. The standard curve was obtained by measuring NO_3_^–^ concentration from standard solutions at concentrations of 0, 1, 5, 10, 25, 50, and 100 ppm NO_3_^–^.

### Microbial Analyses

Nucleic acids were extracted from 0.250 g fresh soil using the DNeasy PowerSoil Kit (Qiagen, Hilden, Germany) according to the manufacturer’s recommendation. DNA quality and quantity were analyzed by UV/Vis spectrophotometry with the QIAxpert System (Qiagen, Hilden, Germany).

The abundance of specific functional groups involved in the N cycle was assessed using a SYBR Green-based qPCR approach. To quantify AOA and AOB, the nitrification gene *amoA* was used as a molecular marker (Bru et al., 2011), whereas the denitrifiers *nirK* and *nirS* genes as well as *nosZ*-I and *nosZ*-II genes were used to target NO_2_^–^ and N_2_O reducers, respectively (Bru et al., 2011; Jones et al., 2013). Potential amplification inhibition by extraction contaminants was tested across all samples using a qPCR assay of pGEM-T plasmid (GenBank^®^ Accession No. X65308; Promega, Madison, WI, United States) spiked into the soil DNA at equimolar concentration in all samples and using the plasmid specific primers SP6 and T7 for PCR. The qPCR reactions were performed in a final volume of 20 μl using the primers listed in Table 2. All the reactions (except for *nosZ*-II) were performed with 1 or 2 μM of each primer, 1X SSO Advanced™ Universal SYBR^®^ Green Supermix (Bio-Rad Laboratories, Hercules, CA, United States) and 5 ng of template DNA. For nosZ-II, 1X Takyon low ROX SYBR 2X MasterMix blue dTTP (Eurogentec, Seraing, Belgium) was used. The cycling conditions for all reactions consisted of a polymerase activation step at 98°C for 3 min, followed by denaturation at 95°C for 15 s, annealing at primer-specific temperatures (see Table 2) for 30 s, and elongation at 72°C for 15 s. Those last three steps were repeated between 35 and 40 times, depending on the targeted gene with or without a touchdown approach (see Table 2). Melting curves were generated by increasing the temperature from 75 to 95°C by 0.5°C every 5 s at the end of the amplification cycles in order to verify the amplification specificity. All reactions were done with a thermocycler CFX Connect™ Real-Time System (Bio-Rad Laboratories, Hercules, CA, United States), and the results were recorded and analyzed using the CFX Maestro software (Bio-Rad Laboratories, Hercules, CA, United States). The DNA standards were prepared from purified PCR products obtained by amplifying the targets from a pool of DNA from all samples. The concentrations used for the standard curves ranged from 10^–2^ to 10^–7^ng of DNA per reaction. The qPCR efficiencies (E) ranged between 99.0 and 110.4%.

**TABLE 2:** Primers used for the qPCR.

Genes	Primer sequences	References	Annealing temperatures	Cycle numbers
*16S*	515F: GTGCCAGCMGCCGCGGTAA 806R: GGACTACNVGGGTHTCTAAT	Frey et al., 2016	52°C	35
*amoA (B)*	amoA-1F: GGGGTTTCTACTGGTGGT amoA-2R: CCCCTCKGSAAAGCCTTCTTC	Rotthauwe et al., 1997	55°C	35
*amoA (A)*	CrenamoA19F: ATGGTCTGGCTWAGACG CrenamoA616R: GCCATCCATCTGTATGTCCA	Leininger et al., 2006; Tourna et al., 2008	55°C	35
*nirK*	nirK 876F: ATYGGCGGVCAYGGCGA nirK 1040R: GCCTCGATCAGRTTRTGGTT	Henry et al., 2004	63°C with −1°C per cycle	6
			58°C	30
*nirS*	nirS CD3aF Throback: GTSAACGYSAAGGARACSGG nirS R3cd Throback: GASTTCGGRTGSGTCTTSAYGAA	Throbäck et al., 2004	63°C with −1°C per cycle	6
			58°C	30
*nosZ-I*	nosZ1840F: CGCRACGGCAASAAGGTSMSSGT nosZ2090R: CAKRTGCAKSGCRTGGCAGAA	Henry et al., 2006	65°C with −1°C per cycle	6
			60°C	30
*nosZ-II*	nosZ-II-F: CTIGGICCIYTKCAYAC nosZ-II-R: GCIGAICARAAITCBGTRC	Jones et al., 2013	54°C	40

Overall differences in bacterial community structure were assessed using a metabarcoding approach. The PCR amplification of the bacterial and archaeal 16S rRNA gene (V3–V4 region) was performed on 20 ng of template DNA using 0.4 μM of each primer, 341F and 806R (Frey et al., 2016), with 1X of GoTaq^®^ Colorless Master Mix from Promega (Promega, Madison, WI, United States) in a final volume of 25 μl. PCR amplification was carried out in technical triplicates, and products were pooled prior to sequencing. Cycling conditions for the PCR reactions consisted of a polymerase activation step at 95°C for 5 min, followed by denaturation at 95°C for 40 s, annealing at 58°C for 40 s, and elongation at 72°C for 1 min. Those last three steps were repeated 30 times. PCR products were sent to the Functional Genomics Center Zurich (FGCZ, Zurich, Switzerland) for indexing PCR. Indexed PCR products were purified, quantified, and pooled in equimolar ratios before pre-sequencing on the Illumina MiniSeq platform (Illumina, San Diego, CA, United States) to inform library re-pooling for achieving optimal read count distribution across samples. Final sequencing was done on the Illumina MiSeq platform using the v3 chemistry for PE300 reads.

### Bioinformatics

Sequence data were processed using a customized pipeline largely based on VSEARCH (Rognes et al., 2016) as previously described (Longepierre et al., 2021). Briefly, PCR primers were trimmed using CUTADAPT (Martin, 2011), allowing for one mismatch and filtered for PhiX contamination by running the reads against the PhiX genome (accession NC_001422.1) using Bowtie2 (Langmead and Salzberg, 2012). Trimmed paired-end reads were merged using the *fastq_mergepairs* function in VSEARCH and quality-filtered using the *fastq_filter* function in VSEARCH with a maximum expected error of one (Edgar and Flyvbjerg, 2015). Sequences were dereplicated using the *derep_fulllength* function in VSEARCH and delineated into amplicon sequence variants (ASVs) using the UNOISE algorithm (Edgar, 2016c) implemented in VSEARCH with an alpha of 2 and a *minsize* of 4. Potentially chimeric ASV sequences were identified and removed using the UCHIME2 algorithm (Edgar, 2016b) implemented in VSEARCH. The remaining ASV sequences were tested for the presence of ribosomal signatures using Metaxa2 (Bengtsson-Palme et al., 2015) and unsupported sequences were discarded. The final ASV table was obtained by mapping the quality-filtered reads of each sample against the verified ASV sequences using the *usearch_global algorithm* in VSEARCH with the settings maxrejects 300, maxaccepts 0, maxhits 1, and a minimum identity of 97%. The taxonomic classification of each verified ASV sequence was performed by running the SINTAX algorithm (Edgar, 2016a) implemented in VSEARCH against the SILVA v.132 database (Pruesse et al., 2007) using a bootstrap cut-off of 0.8. ASVs not assigned at the domain level of bacteria or archaea, as well as ASVs assigned to organelle structures (chloroplasts, mitochondria), were removed from the ASV table. Raw sequences were deposited in the European Nucleotide Archive under the accession number PRJEB48039.

### Statistical Analysis

All statistical analyses were performed in R (R Core Team, 2017), and *P*-values < 0.05 were considered significant in all statistical tests. Pots where plants failed to grow were removed from the analysis in order to assess compaction resilience under pea or wheat growth, leading to a total of 71/80 pots and 96/100 pots for pea and wheat, respectively, with a minimum of three replicates per condition. Moreover, for some chemical parameters, additional pots considered as outliers were removed. To remove outliers, the quantiles of 0.25 and 0.75 of the measured parameters were calculated within compaction condition (e.g., control, light, moderate, and severe conditions) and extreme pot values outside the quantile range were discarded, but at least three replicates per condition were always kept. Furthermore, both crops were analyzed separately to account for intrinsic differences between the different crops, i.e., wheat and pea.

Univariate properties including plant biomass, physical parameters, chemical parameters, and the estimated copy numbers of each gene were analyzed with non-parametric tests since the normality of residuals and the homogeneity of variance were statistically not supported. Compaction effects within each sampling time were assessed using the Kruskal–Wallis test, followed by the Dunn’s *post hoc* test (Dinno, 2017) implemented in the R packages *stats (v.4.0.4)* and *dunn.test (v1.3.5)*, respectively. The plant biomass for both pea and wheat were presented as percent change in comparison to the respective uncompacted controls. Spearman correlations among chemical parameters and plant biomass were performed with the function *corr.test* from the R package *stats*.

The overall concentrations of soil CO_2_, N_2_O, NH_4_^+^, and NO_3_^–^ were integrated over the entire course of the experiment. Variations in CO_2_, N_2_O, NH_4_^+^, and NO_3_^–^ concentrations between each compaction condition were compared using the Kruskal–Wallis test, followed by Dunn’s *post hoc* test (Dinno, 2017).

Sequencing depth was examined using barplots and rarefaction curves with the *rarecurve* function from the *vegan* package (Oksanen et al., 2020). In order to account for differences in sequencing depth (Supplementary Figure 1), differences in α-diversity (observed richness, Pielou’s evenness, and Shannon diversity) and β-diversity (Bray–Curtis dissimilarity) were determined from 50 iteratively subsampled and square root-transformed ASV count tables (Martiny et al., 2017; Hemkemeyer et al., 2019) using the *rrarefy*, *specnumber*, *diversity*, and *vegdist* functions in *vegan.* The effects of compaction, crop, and time on α-diversity were assessed using univariate permutational analysis of variance (PERMANOVA; Anderson, 2001) as implemented in the *adonis* function from *vegan* using 9,999 permutations. Pairwise tests between factor levels were performed using the *pairwise.perm.manova* function implemented in package *RVAideMemoire* v.0.9-73 (Hervé, 2020). The temporal evolution of the treatment effects, as well as the influence of soil chemical properties and plant biomass on microbial β-diversity, were assessed using multivariate PERMANOVA *via* the *adonis* function with 9,999 permutations. The differences in β-diversity between compaction conditions and crop were assessed by constrained ordination using the canonical analysis of principal coordinates (CAP; Anderson and Willis, 2003) implemented as the *CAPdiscrim* function in the *BiodiversityR* package (Kindt and Coe, 2005) with 9,999 permutations. In addition, factors labeled as significant in the PERMANOVA test were also used as a constraining factor in order to build a parsimony model using *ordistep* in *vegan* and displayed by distance-based redundancy analysis (db-RDA; Legendre and Andersson, 1999), using *dbrda* in *vegan* for each crop.

The response of individual taxa to soil compaction for each crop was assessed using PERMANOVA *via* the *adonis* function with 9,999 permutations. Adjustments for multiple testing were performed using *q*-values (Storey and Tibshirani, 2003) implemented in the *qvalue* function of the R package *qvalue* v.2.16.0 (Storey et al., 2020), and *q*-values < 0.1 were considered significant. To avoid the inflation of Type II error due to the impact of rare taxa on multiple testing corrections, ASVs with an overall abundance less than 0.0005% (around 20% of the ASVs) were removed. The taxonomic trees for the prokaryotic ASVs assigned at the genus level showing a significant increase or decrease in their relative abundance under compaction treatments when compared to the control for pea or wheat were generated with iToL v6.1.2 (Letunic and Bork, 2019) based on a tree matrix retrieved from the taxonomy table using the *taxa2dist* function from the *vegan* package and the *hclust* function from the *ade4* package, respectively. In order to make inferences with respect to the potential lifestyle of the taxa for prokaryotes, literature searches were performed, which were supported by the literature also available through Faprotax v1.2.4 (Louca et al., 2016).

## Results

### Soil Bulk Density

**FIGURE 1:**
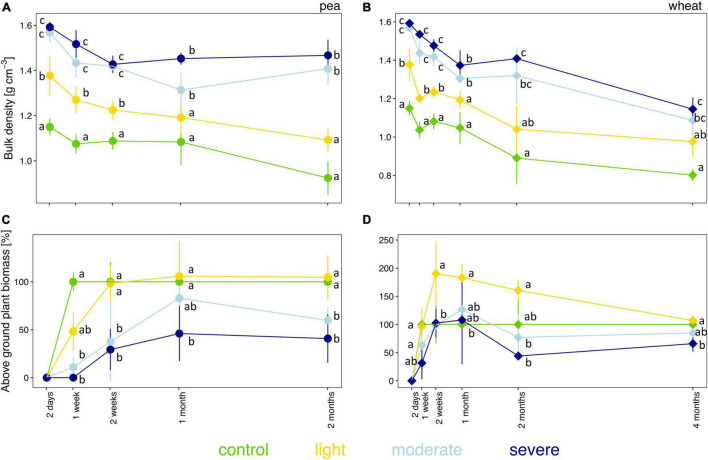
Temporal evolution of bulk density **(A,B)** and plant biomass **(C,D)** between the different compaction conditions over 2 months for pea **(A,C)** and 4 months for wheat **(B,D)**. Different letters indicate significant differences between the compaction levels at each time point as obtained by Kruskal–Wallis and the Dunn’s *post hoc* test.

The initial severity of soil compaction, estimated by measuring soil bulk density 2 days after compaction, depended on the initial soil moisture level (Figures 1A,B and Supplementary Figure 2A). Two days after compaction, bulk densities in the light, moderate, and severe compaction treatments were significantly higher than in the uncompacted control (Figures 1A,B and Supplementary Figure 2A). Moreover, the two moist conditions (i.e., moderate and severe compaction) had significantly higher bulk densities than the light compaction but showed no difference between each other. A strong positive correlation (rho = 0.81) was found between bulk density and soil moisture content for the initial compaction impact (Supplementary Figure 2B) and was maintained during the whole experiment with rho = 0.76 for pea (Supplementary Figure 3A) and rho = 0.64 for wheat (Supplementary Figure 3B). Over time, bulk density did not fully recover for all compacted conditions under pea or wheat with a significant difference of the moderate and severe treatment compared to the uncompacted control for pea and wheat (Figures 1A,B).

### Plant Biomass

The fresh biomass of pea and wheat were influenced by the degree of soil compaction (Figures 1C,D). Pea biomass was lower than control under moderate and severe compaction after 1 week of growth and onward (Figure 1C). From the second week onward, pea biomass under light compaction recovered and had similar biomass as the uncompacted control (Figure 1C). Moreover, there was a strong negative correlation (rho = −0.80) between bulk density and pea biomass (Supplementary Figure 3C).

Wheat biomass remained lower under severe compaction compared to control (Figure 1D). After 2 weeks and up to 2 months of growth, wheat biomass was significantly higher under light compaction compared to the other treatments. The negative correlation between bulk density and wheat biomass was lower than the one found for pea biomass (rho = −0.29, Supplementary Figure 3D).

Overall, wheat tended to be less sensitive than pea to compaction (Figures 1C,D). Soil compaction also led to a greater variability in plant germination and delayed germination in some pots without any observable plant growth under moderate (2 out of 5) and severe (5 out of 5) compaction for pea after 1 week (Supplementary Table 1). Furthermore, plant productivity decreased as no seeds or ears were produced for pea and wheat, respectively, under moderate and severe conditions (Supplementary Table 1).

### Soil Pore CO_2_ and N_2_O Concentrations

**FIGURE 2:**
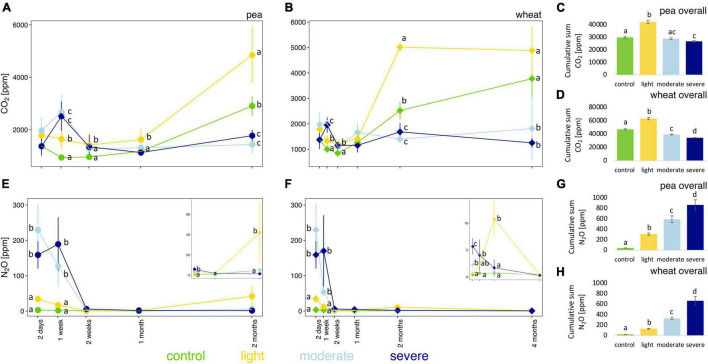
Temporal evolution and cumulative values of soil CO_2_ **(A–D)** and N_2_O **(E–H)** concentrations between the different compaction conditions over 2 months for pea **(A,C,E,G)** and 4 months for wheat **(B,D,F,H)**. Different letters indicate significant differences between the compaction levels as obtained by Kruskal–Wallis and Dunn’s *post hoc* tests.

The initial impact of compaction, measured 2 days after the compaction event and before plant growth, did not significantly change the soil pore CO_2_ concentration; only after 1 week, CO_2_ concentrations are higher under moderate and severe compaction compared to light compaction and control (Figures 2A,B). However, after 1 month for pea and 2 months for wheat, the soil pore CO_2_ concentration was much higher under light compaction and in the control (Figures 2A,B) than under moderate and severe compaction. The cumulative soil pore CO_2_ concentrations for the entire experiment were the highest under light compaction compared to the rest of the treatments (Figures 2C,D). The soil pore CO_2_ concentration correlated stronger with pea (rho = 0.81) or wheat (rho = 0.70) biomass than with the different compaction conditions (Supplementary Figure 4).

Compaction significantly increased soil pore N_2_O concentrations under moderate and severe compaction compared to light compaction and control in the first week (Figures 2E,F and Supplementary Figure 5). Soil pore N_2_O concentrations in the compacted soils slowly returned to the values of the uncompacted control soils and were ultimately not significantly different between the compaction conditions for both pea and wheat (Figures 2E,F). The cumulative soil pore N_2_O concentration for the entire experiment was the highest under severe compaction, followed by moderate and light compaction and the uncompacted control with higher concentrations under pea than wheat (Figures 2G,H).

### Soil NH_4_^+^ and NO_3_^–^ Concentrations

**FIGURE 3:**
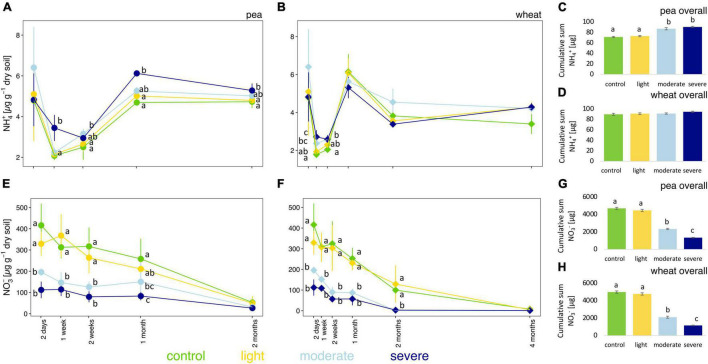
Temporal evolution and cumulative values of soil NH_4_^+^ **(A–D)** and NO_3_^–^ **(E–H)** concentrations between the different compaction conditions over 2 months for pea **(A,C,E,G)** and 4 months for wheat **(B,D,F,H)**. Different letters indicate significant differences between the compaction levels conditions at each time point as obtained by Kruskal–Wallis and Dunn’s *post hoc* tests.

After 2 days, moderate and severe compaction significantly decreased soil NO_3_^–^ concentrations (Figures 3E,F) compared to the control but had no impact on NH_4_^+^ concentrations (Figures 3A,B). Over the entire experiment, however, soil NH_4_^+^ concentrations were higher in compacted soils compared to the control for pea (Figures 3A–C) with a clear positive correlation (rho = 0.60) between bulk density and soil NH_4_^+^ concentrations (Supplementary Figure 6A). However, for wheat, no significant changes in NH_4_^+^ concentrations were observed from 2 weeks onward (Figures 3B–D) and it translated into a weaker positive correlation (rho = 0.27) between the soil bulk density and soil NH_4_^+^ concentrations (Supplementary Figure 6B).

Soil NO_3_^–^ concentrations were significantly lower up to 1 month for pea and up to 2 months for wheat under moderate and severe compaction compared to the uncompacted control (Figures 3E,F). Negative correlations between bulk density and NO_3_^–^ soil concentrations were found for pea (rho = −0.69) and wheat (rho = −0.79), respectively (Supplementary Figures 7A,B). Soil NO_3_^–^ concentrations correlated negatively with pea (rho = −0.57) and wheat (rho = −0.66) biomass (Supplementary Figures 7C,D). The cumulative soil NO_3_^–^ concentrations were the lowest under severe compaction, followed by moderate and light compaction and the uncompacted control for pea and wheat (Figures 3G,H).

### Microbial Community Analysis

#### Soil Microbial Functional Genes

**FIGURE 4:**
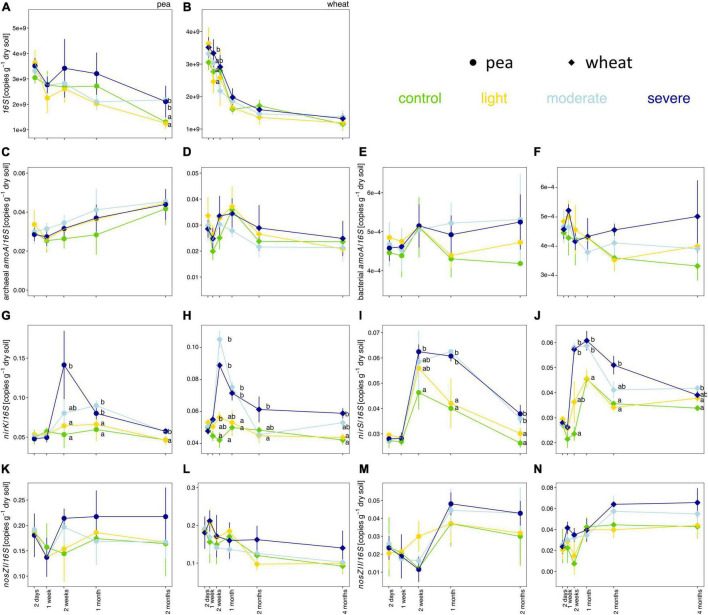
Temporal evolution of the total estimated copy numbers of the 16S rRNA gene **(A,B)** as well as the relative estimated copy numbers (copies per 16S rRNA gene) of the archaeal *amoA* **(C,D)** bacterial *amoA* **(E,F)**, *nirK* **(G,H)**, *nirS* **(I,J)**, *nosZ-I* **(K,L)**, and *nosZ-II* **(M,N)** genes per gram of dry soil between the different compaction conditions over 2 months for pea **(A,C,E,G,I,K,M)** and 4 months for wheat **(B,D,F,H,J,L,N)**. Different letters indicate significant differences between the compaction levels conditions at each time point as obtained by Kruskal–Wallis and Dunn’s *post hoc* tests.

Soil compaction did not consistently change the copy numbers per gram of dry soil for the 16S rRNA gene as well as the proportions of *bacterial amoA*, archaeal *amoA*, *nirS*, *nirK*, *nosZ*-I, and *nosZ-II* genes in the bacterial community (Figure 4). The estimated copy number per gram of dry soil for the 16S rRNA gene tended to be higher under moderate and severe compaction under pea compared to wheat (Figures 4A,B). The ratios of *nirS* and *nirK* to 16S rRNA genes were significantly higher under severe compaction and partially under moderate compaction from 2 weeks post-compaction onward for both pea and wheat (Figures 4G–J). The ratios of *bacterial amoA*, archaeal *amoA*, *nosZ*-I, and *nosZ-II* genes to 16S rRNA genes did not significantly change under compaction for both pea and wheat (Figures 4C–F,K–N).

#### Soil Microbial Community Structure

**FIGURE 5:**
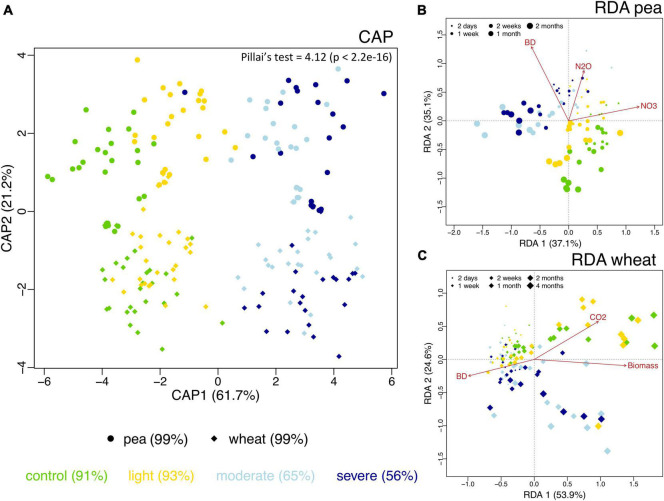
Differences in soil bacterial community structure across the two different plant systems (pea, circles; wheat, diamonds) and the unplanted initial impact (squares) under the different compaction levels including the uncompacted control (green) as well as the light (yellow), moderate (light blue) and severe (dark blue) compaction treatments as assessed by canonical analysis of principal coordinates (CAP) based on Bray–Curtis dissimilarities **(A)**. The CAP reclassification success rates are provided in parentheses next to each treatment group and provide a quantitative estimation of the degree of discrimination between the treatment groups. The CAP equivalent to Pillai’s trace test (with *p*-value in brackets) is provided in the upper-right corner and provides an indication of the overall effect size. Bacterial community structure and its relationship with time since compaction and the measured chemical parameters for pea **(B)** and wheat **(C)** as assessed by distance-based redundancy analysis (dbRDA).

The observed richness of the prokaryotic community significantly increased under compaction (light, moderate, and severe) for both pea and wheat (Supplementary Figure 8A). Pielou’s evenness significantly decreased under moderate and severe compaction under pea but remained comparable across compaction treatments under wheat (Supplementary Figure 8B). Shannon diversity did not show any differences due to soil compaction (Supplementary Figure 8C). Overall, there seemed to be a maximum alpha diversity under light compaction. Many factors were shaping the bacterial community structure (Table 3). The plant (explaining 10% of the variance) bulk density (16%) had strong effects on prokaryotic community structures. Each crop and compaction treatment harbored a statistically (*p* < 0.033) distinct microbial community, except between moderate and severe compaction within each crop (*p* < 0.285), as demonstrated by the high CAP reclassification success rates of 99% for the crop and 56–93% for the compaction conditions (Figure 5A). Lower reclassification rates under moderate and severe compaction were attributed to the smaller differences of the microbial communities between those two conditions. The interaction between crop and bulk density (15%) as well as the temporal component (34%) also influenced the prokaryotic community structure (Table 3 and Figures 5B,C). The community structure under pea was mainly driven by time, bulk density, and soil NO_3_^–^ and N_2_O concentrations, whereas under wheat, it was mainly driven by time, bulk density, plant biomass, and CO_2_ concentrations (Figures 5B,C).

**TABLE 3:** Effects of soil compaction, plant, and soil chemical properties on microbial community structure.

	*F* (*P*)*^a^*	*R*^2^ ^b^
Plant (P)	2.19 (<0.001)	0.010
Bulk density (BD)	3.30 (<0.001)	0.016
Moisture content (MC)	2.90 (0.004)	0.013
Plant biomass	3.98 (<0.001)	0.019
CO_2_ in soil	1.55 (<0.001)	0.007
N_2_O in soil	1.99 (0.007)	0.009
NO_3_^–^ in soil	1.24 (<0.001)	0.006
NH_4_^+^ in soil	1.74 (0.049)	0.008
Time	1.48 (<0.001)	0.034
P × BD	1.01 (0.001)	0.015
P × MC	0.38 (1.000)	0.002
BD × MC	1.55 (<0.001)	0.007
P × BD × MC	0.43 (1.000)	0.002
Residuals		0.86

*^a^Values indicate the F-ratio (F) and the level of significance (P) assessed by PERMANOVA.*

*^b^Values indicate the explained variance (R^2^) assessed by PERMANOVA.*

#### Compaction-Sensitive Microbial Taxa

After correcting for multiple testing, around 3% (1% assigned at genus level) out of the 17,262 prokaryotic ASVs under pea and around 4% (2% assigned at genus level) out of the 17,412 prokaryotic ASVs under wheat responded significantly to soil compaction. Most of these sensitive ASVs (increasing or decreasing under compaction) were unique for a specific crop, and only very few ASVs (65) were responding universally across both crops (Supplementary Figure 9).

**FIGURE 6:**
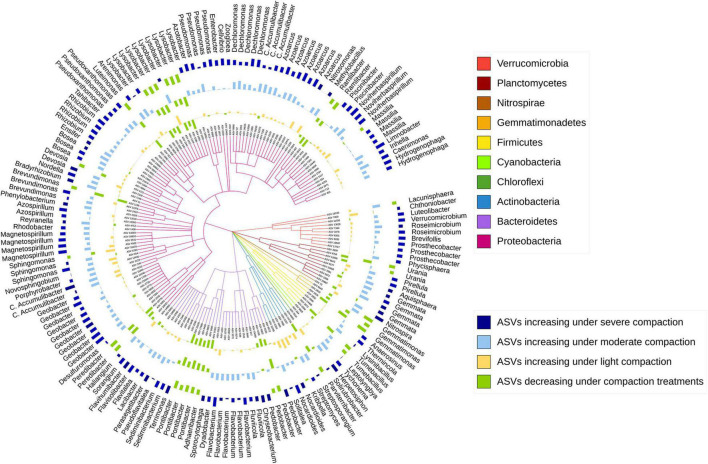
Taxonomic tree showing the bacterial ASVs assigned at the genus level and responding significantly to compaction (PERMANOVA, *q* < 0.1) for pea. The barplots show the *z*-transformed relative abundances of these ASVs, with yellow, light-blue, and dark-blue bars representing ASVs relatively enriched under the light, moderate, and severe compaction treatments, respectively, and the green bars representing the ASVs relatively enriched under the control conditions.

**FIGURE 7:**
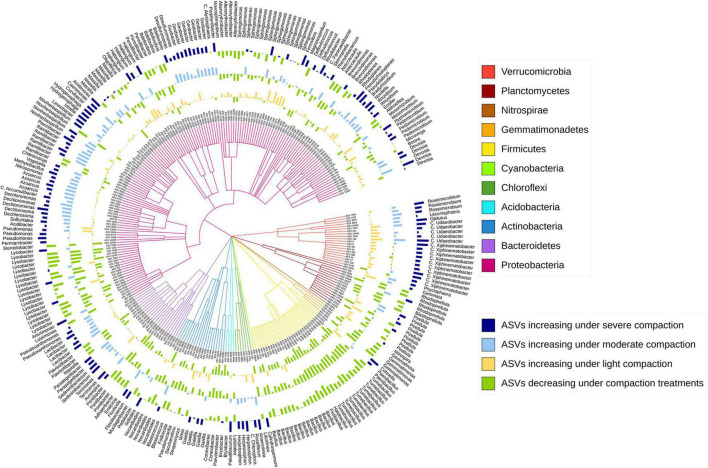
Taxonomic tree showing the bacterial ASVs assigned at the genus level and responding significantly to compaction (PERMANOVA, *q* < 0.1) for wheat. The barplots show the *z*-transformed relative abundances of these ASVs, with the yellow, light-blue, and dark-blue bars representing ASVs relatively enriched under the light, moderate, and severe compaction treatments, respectively, and the green bars representing the ASVs relatively enriched under the control conditions.

The sensitive ASVs, assigned at a genus level, were broadly spread across the taxonomic tree and present in all major bacterial phyla (Figures 6, 7). Some ASVs with contrasting responses to soil compaction were assigned to the same phylum and/or genus (Figures 6, 7). Salient examples of bacterial genera with ASVs increasing under compaction for pea (Figure 6) and wheat (Figure 7) included *Ensifer, Pseudoxanthomonas, Azoarcus, Vogesella*, *Pseudomonas, Dechlorosoma, Rhodobacters, Dechlorosomas, Desulfuromonas, Geobacter, Azospirillum, Magnetospirillum, Bradyrhizobium, Rhizobium, Devosia, Ramlibacter, Massilia*, and *Nitrosomonas* (all *Proteobacteria*), *Flavobacterium, Lacibacter* and *Terrimonas* (*Bacteroidetes*), *Lacunisphaera, Luteolibacter* and *Opitutus* (*Verrucomicrobia*), *Gemmata* and *Pirellula* (*Planctomycetes), Nitrospira* (Nitrospirae), *Nocardioides* (*Actinobacteria*), and *Anaerolinea* (*Chloroflexi*). Conversely, genera with ASVs showing higher relative abundance in the uncompacted control plots included *Tumebacillus* and *Bacillus* (*Firmicutes*), *Altererythrobacter, Lysobacter* (*Proteobacteria), Adhaeribacter, Pedobacter, Pontibacter* and *Flavisolibacter* (*Bacteroidetes*), and *Conexibacter (Actinobacteria)*. No archaeal ASVs showed a significant response to compaction. In addition to the ASV level response, these shifts in relative abundance were also statistically evaluated by aggregating the data at all assigned taxonomic levels from genus to phylum (Supplementary Data 1).

## Discussion

### Soil Bulk Density

As hypothesized, the degree of soil compaction depended on the initial soil moisture content (Figures 1A,B and Supplementary Figures 2A,B). As already demonstrated in previous studies, soil compaction, approximated by the bulk density, is more severe at higher soil moisture contents (Smith et al., 1997; Hartmann et al., 2014), which was confirmed in our study. However, if all soil pores are filled with water, the soil cannot be compacted any further (Smith et al., 1997). This might explain why we did not observe significant differences in bulk densities between moderate and severe compaction. Alternatively, the threshold of soil elasticity for this specific type of soil under this specific applied pressure may have been reached, and thus, the soil could not have been compacted any further (Horn et al., 1995). Soil bulk density did not entirely recover over the course of 2–4 months under either crop even under light compaction (Figures 1A,B). The continuous decline in the bulk density of the control treatment was caused by root growth. Although not quantified, the root systems for both pea and wheat tended to be less developed with thinner and have less deep roots under compaction, as demonstrated in other studies (Unger and Kaspar, 1994). Using plant rooting activity to restore compacted soils is a well-accepted approach, but not all crops are suitable for this because of their thin and shallow roots (Drewry, 2006; Jabro et al., 2021). Additionally, such an approach typically requires more than one growing season as the natural recovery process can take decades (Drewry, 2006; Jabro et al., 2021) when compared to mechanical loosening through, for example, deep ripping or disking (Nawaz et al., 2013). Furthermore, recovery is largely dependent on the physical and mineralogical constitution of the soil (Schjønning et al., 2015), but here, we tested only one type of soil and we can therefore not assess the influence of these factors.

### Plant Biomass and Soil Respiration

In general, an increase in soil bulk density due to compaction reduces stem growth and leaf area, resulting in reduced plant biomass accumulation (Nawaz et al., 2013; Shah et al., 2017). It can also delay or prevent germination, as observed for pea under moderate and severe compaction (Nawaz et al., 2013; Shah et al., 2017). Here, soil compaction reduced pea biomass production more severely than wheat (Figures 1C,D and Supplementary Table 1). It has been documented that soil compaction effects are plant specific and often affect legumes more than cereals potentially because of their symbiosis with microbes that can also be affected by compaction (Batey, 2009; Siczek and Lipiec, 2011; Arvidsson and Håkansson, 2014). However, known symbiotic diazotrophs such as *Rhizobium* and *Bradyrhizobium* increased in relative abundance in our study, thus not confirming this hypothesis. But those symbiotic diazotrophs were sampled in the bulk soil and may not entirely reflect the abundance of symbiotic nitrogen-fixing bacteria in the root system as well as their nitrogenase activities.

Usually, reduced soil porosity decreases the uptake of nutrients due to restricted root growth, leading to biomass losses (Rosolem et al., 2002). It has further been demonstrated that under such unfavorable conditions, the plant’s effort to develop its root system and accumulate biomass can reduce fruit production even though the total plant biomass remains comparable between compacted and uncompacted soils (Colombi and Keller, 2019). The latter is also demonstrated in our study for both pea and wheat (Supplementary Table 1).

Nevertheless, under light compaction, wheat biomass tended to be higher compared to the control (Figures 1C,D). Compaction can also, to some extent, increase contact between roots and soil particles, which may lead to faster ion exchange between the soil matrix and roots, and can therefore improve nutrient accessibility and thus plant growth (Nawaz et al., 2013).

At the same time, soil respiration, approximated by measuring soil pore CO_2_ concentrations, correlated mostly with crop biomass (Figures 2A,B and Supplementary Figures 4A,B), indicating an increase in root respiration with the development of the plant root system (Aerts et al., 1991). However, soil CO_2_ concentrations under moderate and severe compaction were initially comparable to the control and stayed quite stable over time (Figures 2C,D); this was surprising since soil compaction generally reduces aerobic niches and therefore soil CO_2_ respiration. The lack of reduction in soil pore CO_2_ concentration over the experiment in all compacted treatments suggests the presence of aerobic niches and that total soil anaerobicity might not have been achieved even under severe compaction.

### Parameters Associated With the Nitrogen Cycle

Under moderate and severe compaction, we observed higher cumulative soil pore N_2_O concentrations (Figures 2G,H) as well as a decrease in soil NO_3_^–^ (Figures 3E–H and Supplementary Figures 7A,B). Additionally, denitrifier abundances estimated *via nirS, nirK*, *nosZ*-I, and *nosZ*-II gene copy numbers tended to increase under severe compaction and partially under moderate compaction when compared to the control (Figures 4G–N). All those results combined indicate increased denitrifier activity, which is in line with other studies (Pupin et al., 2009; Bao et al., 2012). Indeed, soil compaction can reduce oxygen availability and promote denitrification in soil (Pupin et al., 2009). Incomplete denitrification can subsequently increase N_2_O production in particular after the additional application of N fertilizer (Bao et al., 2012) and soil moisture levels favoring denitrification (Ruser et al., 2006). Since *nir*S/K copies significantly increased under compaction while *nos*Z-I/II copies were not significantly higher (Figure 4), it can be speculated that a higher N_2_O production coupled to a largely unchanged N_2_O consumption increased soil pore N_2_O concentrations. However, linking gene abundances with concentrations of substrates (e.g., NO_3_^–^) and products (e.g., N_2_O) remains challenging because of the multi-step nature of denitrification and the dependency of each individual step on many different abiotic (e.g., moisture content, temperature, and pH) and biotic (different microbial taxa involved and competition with plants) factors (Butterbach-Bahl et al., 2013). In fact, denitrifiers are a polyphyletic group with different bacterial taxa involved in the different steps of the denitrification pathway (Hayatsu et al., 2008). Additionally, NO_3_^–^ decreased throughout the experiment, especially under light compaction and in the control (Figures 3E,F and Supplementary Figures 7C,D), which is likely due to its consumption by plants during growth (Beevers and Hageman, 1983; Bao et al., 2012).

In contrast, NH_4_^+^ is primarily metabolized by AOAs and AOBs during nitrification under aerobic conditions or consumed by plants (Beevers and Hageman, 1983). The hypothesized reduction in oxygen availability and the reduced development of the plant root system has likely limited NH_4_^+^ consumption (Bao et al., 2012) and might explain the accumulation of NH_4_^+^ in severe compaction (Figures 3A–D and Supplementary Figure 6). However, the proportion of estimated archaeal and bacterial *amoA* gene copy numbers per gram of soil did not differ significantly in compacted soils compared to the control (Figures 4C–F). A previous study has shown that microbial nitrification rates in soil do not begin to decline until bulk densities exceed 1.5 g cm^–3^ (De Neve and Hofman, 2000), which was surpassed only under severe and to some degree also under moderate compaction. A potential reduction of nitrifier activity might therefore explain the accumulation of NH_4_^+^ in these soils. However, there is no clear evidence from *amoA* gene quantification that these conditions induced a reduction of nitrifiers in this experiment. To date, there is no study linking nitrifier abundance to nitrifier activity and nitrification rates under such conditions. Only at very high bulk densities of around 1.7 g cm^–3^ were nitrifier abundances shown to decrease (Pupin et al., 2009), but this threshold was not met in any of the treatments in this experiment. Moreover, comparable soil pore CO_2_ concentrations under all treatments, including the control (Figures 2A,B), indicated that aerobic respiration was not limited. Thus, the lack of a clear shift from aerobic to anaerobic conditions under compaction could explain why the measured oxygen-dependent functional guilds did not change significantly.

### Soil Microbial Diversity

The estimated copy numbers of 16S rRNA genes per gram of soil remained comparable between all compaction treatments (Figures 4A,B). A previous study showed that bacterial biomass decreased only when bulk density reached the critical threshold of 1.6 g cm^–3^ (Li et al., 2002). In this experiment, such values were obtained only under the severe compaction and not consistently, which could explain the lack of impact on microbial biomass (Figures 3A–B).

Even though the bacterial biomass did not change, the soil bacterial community structure shifted due to compaction and was mainly driven by crop, bulk density, moisture content, and time after compaction (Figure 5). It is well accepted that different crops recruit different bacterial communities (Grayston et al., 1998) and that soil compaction induces changes in bacterial community structures through altering the soil’s physicochemical properties (Hartmann et al., 2014; Longepierre et al., 2021) as well as soil moisture (Drenovsky et al., 2004). Moreover, an increase in microbial richness due to soil compaction, as observed in a previous soil compaction study in forests (Hartmann et al., 2014), was also observed in our experiment but was statistically not well supported (Supplementary Figure 8).

Shifts in the bacterial community structure correlated with soil NO_3_^–^/N_2_O/CO_2_ concentrations (Figures 5B,C). The concentrations of these elements in soil can be driven by oxygen availability (Bao et al., 2012). As shown in previous studies, oxygen limitation due to increased bulk density under compaction is one of the main drivers of bacterial community structure. Indeed, bacterial species capable of metabolizing under a low partial pressure of oxygen commonly thrive under these conditions (Hartmann et al., 2014). Bacterial genera with known anaerobic lifestyles such as *Desulfuromonas, Geobacter, Anaerolinea, Anaerosinus, Dechlorosoma, Dechlorosomas, Pseudoxanthomonas, Vogesella*, *Pseudomonas, Azoarcus, Pirellula, Opitutus, Lacunisphaera, Ensifer, Rhodobacter, Luteolibacter, Gemmata, Lacibacter*, and *Terrimonas* significantly increased in relative abundance in the compacted soils compared to the control (Figures 6, 7). Many of these bacterial taxa have been previously shown to increase in compacted arable (Longepierre et al., 2021) and forest soils (Hartmann et al., 2014), paddy rice soils (He et al., 2019), temporarily water-logged fields (Gschwend et al., 2020), sediments (Jackson and Weeks, 2008), and anaerobic bioreactors (Gan et al., 2019), and thus might serve as indicators of oxygen-limited soil environments. Moreover, some of these bacterial taxa, such as *Vogesella*, *Pseudoxanthomonas, Ensifer, Rhodobacter, Decholorosoma*, and *Dechloromonas* are known to be potentially involved in denitrification (Torres et al., 2018; Gan et al., 2019); however, taxonomic information alone is not a reliable measure of functional assignments. Nevertheless, in addition to the partially increased abundances of the *nirS*, *nirK*, *nosZ*-I, and *nosZ*-II genes (Figure 4), the increased relative abundance of those specific taxa may additionally support the hypothesis of increased denitrification under compaction. Other taxa that increased under compaction such as *Pseudomonas*, *Azoarcus, Rhizobium, Bradyrhizobium, Magnetospirillum*, and *Azospirillum* (Bazylinski et al., 2000; Collavino et al., 2014; Li et al., 2017) are potentially involved in N fixation, which is also an oxygen-sensitive pathway and takes place under anaerobic conditions (Limmer and Drake, 1996). The increase of potential diazotrophs may also explain the accumulation of NH_4_^+^ in soil as observed in this experiment (Figures 3A,B), but again, functional inference based on taxonomic assignments is strongly limited.

Analogously, many taxa with aerobic lifestyles can be restricted in compacted soils (Tan and Chang, 2007). In this study, bacterial taxa such as *Tumebacillus, Bacillus, Altererythrobacter, Lysobacter, Flavisolibacter, Pontibacter, Adhaeribacter, Conexibacter*, and *Pedobacter* are known to exhibit a strictly aerobic lifestyle (Mau et al., 2015) and significantly decreased in relative abundance in the compacted soils (Figures 6, 7). However, taxa potentially involved in nitrification, such as *Nitrospira* and *Nitrosomonas* (Hayatsu et al., 2008), surprisingly increased in relative abundance under compaction, as well as many other bacterial taxa with largely aerobic lifestyles such as *Flavobacterium, Ramlibacter, Massilia, Devosia*, and *Nocardioides*. Their presence in compacted soils supports the statement made earlier that aerobic niches were not eliminated by the compaction treatments in this experiment; however, their increase is unexpected, and to date, there is no study to support this finding. Soil compaction induces a multitude of changes to the soil system including alterations of the physical habitat (pores, aggregates), predation by protozoa, and nutrient flows, making it difficult to explain compositional shifts solely based on oxygen limitation. Therefore, strong competition for pore space, nutrient availability, and oxygen can occur, and different bacterial taxa may adapt to these conditions through a variety of strategies such as dormancy and sporulation or changes in their metabolism (van der Linden et al., 1989; Hartmann et al., 2014; Song et al., 2017; Longepierre et al., 2021).

## Conclusion

A single soil compaction event at three different soil moisture levels was sufficient to decrease pea (legume) and wheat (cereal) biomass and to alter the soil microbial community and key N cycling processes over approximately one growing season in both crops. A relative increase in anaerobically metabolizing bacteria was associated with increased NO_3_^–^ consumption and N_2_O production *via* enhanced denitrification, which is consistent with previous studies in agricultural fields. Conversely, nitrification metabolism was likely slowed down and resulted in NH_4_^+^ accumulation in the soil; however, aerobic metabolism was not completely restricted, suggesting that aerobic niches continued to exist. This study combined and linked for the first time the data on soil physicochemical properties, microbial community structure, and soil microbial function as well as plant growth within one experiment. The effects of soil compaction on the two studied biological indicators (e.g., plants and microbes) were not completely aligned. These findings are valuable to better understand the consequences of soil compaction in agriculture in order to formulate more precise regulations for farmers. Further studies across different soil types and spanning a wider range of bulk densities are needed to improve our predictions of how shifts in the soil microbiome after compaction alter the N flow through the system to anticipate the impact of soil compaction on soil functioning that ultimately drives soil fertility and crop production.

## Data Availability Statement

The datasets presented in this study can be found in online repositories. The names of the repository/repositories and accession number(s) can be found below: https://www.ebi.ac.uk/ena, PRJEB48039.

## Author Contributions

ML, MB, and MH conceived the study and designed and initiated the greenhouse experiments. ML and MH operated the greenhouse experiments. ML measured the soil physicochemical properties and plant biomass. MB measured soil pore gas concentrations. ML, RF, DB, and LP performed the microbial analyses. ML, MH, and LP performed the data analyses. ML, RF, and MH wrote the manuscript. All authors contributed to the final manuscript and provided critical feedback.

## Conflict of Interest

The authors declare that the research was conducted in the absence of any commercial or financial relationships that could be construed as a potential conflict of interest.

## Publisher’s Note

All claims expressed in this article are solely those of the authors and do not necessarily represent those of their affiliated organizations, or those of the publisher, the editors and the reviewers. Any product that may be evaluated in this article, or claim that may be made by its manufacturer, is not guaranteed or endorsed by the publisher.
