# Supplementary material for: Mixed Effects of Soil Compaction on the Nitrogen Cycle Under Pea and Wheat
Source: Front Microbiol. 2022 Mar 7;12:822487. doi: 10.3389/fmicb.2021.822487 (PMC8940171; doi:10.3389/fmicb.2021.822487)
Supplement: Supplementary file 1 [file Data_Sheet_1.docx]

Supplementary Figure 1: Total number of amplicon sequence variants (ASVs) per sample and their associated rarefaction curves for bacteria/archaea.

Supplementary Figure 2: (A) Bulk densities two days after compaction. Different letters indicate significant differences between each compaction conditions as obtained by Kruskal-Wallis and Dunn’s post hoc test. (B) Correlation between the initial soil moisture content and the degree of compaction measured by bulk density. Spearman correlation coefficient and p-value are provided in the bottom right corner of the figure.

Supplementary Figure 3: (A, B) Evolution of the bulk density and soil moisture content across the different compaction treatments, i.e., uncompacted control (green), light (yellow), moderate (light blue) and severe (dark blue), over 2 months for pea (A) and over 4 months for wheat (B). Spearman correlation coefficient and p-value are provided in bottom right corner of the figure. (C, D) Correlation between crop yield and bulk density over 2 months for pea (C) and over 4 months for wheat (D). Spearman correlation coefficient and p-value are provided in the bottom right corner of the figure.

Supplementary Figure 4: (A, B) Correlation between soil pore space CO_2_ concentration and bulk density across the different compaction treatments, i.e., uncompacted control (green), light (yellow), moderate (light blue) and severe (dark blue), one week after compaction for pea (A) and wheat (B). Spearman correlation coefficient and p-value are provided in the bottom right corner of the figures. (C, D) Correlation between soil CO_2_ concentration and plant biomass from 1 month to 2 months for pea (C) and to 4 months for wheat (D). Spearman correlation coefficient and p-value are provided in the bottom right corner of the figure.

Supplementary Figure 5: Correlation between soil N_2_O concentration and bulk density across the different compaction treatments, i.e., uncompacted control (green), light (yellow), moderate (light blue) and severe (dark blue), at the beginning of the experiment (e.g., two days and one week after compaction) for pea (A) and wheat (B). Spearman correlation coefficient and p-value are provided in the top left corner of the figures.

Supplementary Figure 6: Correlation between soil NH_4_^+^ concentration in percent and bulk density across the different compaction treatments, i.e., uncompacted control (green), light (yellow), moderate (light blue) and severe (dark blue), over 2 months under pea (A) and over 4 months under wheat (B). Spearman correlation coefficient and p-value are provided in the top left corner of the figures.

Supplementary Figure 7: (A, B) Correlation between soil NO_3_^-^ concentration and bulk density across the different compaction treatments, i.e., uncompacted control (green), light (yellow), moderate (light blue) and severe (dark blue), one week after compaction for pea (A) and wheat (B). Spearman correlation coefficient and p-value are provided in the bottom right corner of the figures. (C, D) Correlation between soil NO_3_^-^ concentration and plant biomass from 1 month to 2 months for pea (C) and to 4 months for wheat (D). Spearman correlation coefficient and p-value are provided in the bottom right corner of the figure.

Supplementary Figure 8: Differences in soil bacteria and archaea observed richness, evenness and Shannon index across the different compaction treatments, i.e., uncompacted control (green), light (yellow), moderate (light blue) and severe (dark blue), within the two plant systems (pea, circles; wheat, diamonds). The data represents the mean (± se) from each condition with n=100 for pea and n= 120 for wheat. The values at the bottom corners indicate the F-ratio (F) with it associated p-value in brackets obtained by univariate permutational analysis of variance (PERMANOVA). The letters indicate significant differences obtained by pairwise PERMANOVA within pea (small letters) and wheat (capital letters).

Supplementary Figure 9: Venn diagram of the statistically significant compaction sensitive bacterial amplicon sequence variants (ASVs) for the two plant systems (e.g., pea and wheat) over all compaction treatments, i.e., light, moderate and severe.
