# Supplementary material for: Mixed Effects of Soil Compaction on the Nitrogen Cycle Under Pea and Wheat
Source: Front Microbiol. 2022 Mar 7;12:822487. doi: 10.3389/fmicb.2021.822487 (PMC8940171; doi:10.3389/fmicb.2021.822487)
Supplement: Supplementary file 2 [file Table_1.docx]

|  | | **1 week** | **2 weeks** | **1 month** | **2 months** | **4 months** | **Seed numbers** | **Seed weight** |
| --- | --- | --- | --- | --- | --- | --- | --- | --- |
| **Peas** | **Control** | 5/5 | 5/5 | 5/5 | 5/5 | - | 63 | 0.74 ($\pm$ 0.17) |
|  | **Light** | 5/5 | 5/5 | 5/5 | 5/5 | - | 74 | 0.66 ($\pm$ 0.08) |
|  | **Moderate** | 3/5 | 3/5 | 3/5 | 5/5 | - | - | - |
|  | **Severe** | 0/5 | 3/5 | 3/5 | 4/5 | - | - | - |
| **Wheat** | **Control** | 5/5 | 5/5 | 5/5 | 5/5 | 5/5 | 3 | 1.2 ($\pm$ 0.12) |
|  | **Light** | 5/5 | 5/5 | 5/5 | 5/5 | 5/5 | 5 | 1.13 ($\pm$ 0.25) |
|  | **Moderate** | 5/5 | 5/5 | 4/5 | 5/5 | 5/5 | - | - |
|  | **Severe** | 3/5 | 4/5 | 5/5 | 4/5 | 4/5 | - | - |

Supplementary Table 1: Assessment of the germination and productivity of pea and wheat.
